# The Toxicological Effects of Emerging Pollutants on Marine Invertebrates: A Review

**DOI:** 10.3390/toxics14050447

**Published:** 2026-05-20

**Authors:** Shenyu Liu, Guangyan Liang, Lei Chen, Shan Wang, Yuxue Qin

**Affiliations:** College of Marine Technology and Environment, Dalian Ocean University, Dalian 116023, China; 15711505696@163.com (S.L.); 13638146296@163.com (G.L.); chenlei@dlou.edu.cn (L.C.)

**Keywords:** emerging contaminants, marine invertebrates, reproductive toxicity, neurotoxicity, combined exposure, toxic pathways

## Abstract

Marine invertebrates are characterized by high species diversity, a wide distribution, ease of culture, low cost, short life cycles and high sensitivity to pollutants, which makes them excellent models for observing toxic effects and elucidating underlying mechanisms. This paper reviews representative species from three phyla—Arthropoda, Mollusca, and Echinodermata—under both single emerging contaminant exposure and combined exposure scenarios, and analyzes the reproductive and neurotoxic impacts of these contaminants on marine invertebrates. Neurotoxicity is mediated by several key mechanisms: inhibition of acetylcholinesterase activity; disruption of neurotransmitter balance, oxidative stress; and cellular damage, interference with embryonic neural development and axis specification, and impairment of neural cell differentiation and migration. Reproductive toxicity impairs reproductive development by disrupting endocrine signaling, inducing oxidative stress, downregulating reproduction-related genes and damaging gonadal structure. Studies have shown that, besides environmental factors, contaminant concentration is closely correlated with toxic potency and differing concentration ratios can lead to either antagonistic or synergistic effects in combined toxicity. Current research has largely focused on single or binary contaminant systems, whereas studies on multi-contaminant mixtures and their interactions with multiple environmental factors remain limited. Future research should prioritize combined exposure to multiple contaminants, long-term multigenerational observations and the development of comprehensive ecological risk assessment models and monitoring standards, thereby providing a scientific basis for marine ecological conservation.

## 1. Introduction

Emerging contaminants are commonly referred to as “new pollutants” in the international scientific literature. This term does not denote a specific class of chemical substances but encompasses a broad range of compounds—including synthetic chemicals, natural products, and microorganisms—that have not yet been systematically incorporated into routine environmental monitoring programs or regulatory frameworks [1]. These contaminants are typically characterized by resistance to biodegradation, potential for long-range environmental transport and a tendency to bioaccumulate in living organisms [2]. Of particular concern is that these substances can elicit adverse biological effects even at trace concentrations, and prolonged exposure may disrupt the endocrine system, impair neurological function, and cause dysregulation of other physiological systems [3,4]. Evidence from Guillotin and Delcourt (2022) has demonstrated associations between persistent organic pollutants (POPs) and a range of health outcomes, including endocrine disorders, neurotoxicity, and various forms of cancer [5]. These contaminants originate from diverse sources, including pharmaceuticals, personal care products, endocrine-disrupting chemicals (EDCs), and industrial or anthropogenic emissions of persistent organic pollutants (POPs) [6,7].

By systematically reviewing a comprehensive body of literature published over the past five years, this paper identifies and summarizes frequently detected emerging contaminants in aquatic environments. These contaminants are broadly classified into four major categories: POPs, EDCs, pharmaceuticals, and microplastics (MPs). POPs encompass short-chain chlorinated paraffins (SCCPs) [8], organometallic compounds such as methylmercury [9], yessotoxins [10], and hexabromocyclododecane (HBCD) [11]. EDCs include chlorinated organophosphate esters such as tris (1-chloro-2-propyl) phosphate (TCPP) and dibutyl phthalate (DBP) [12]. MPs comprise polymethyl methacrylate (PMMA) [13], polystyrene (PS) [10], polypropylene (PP), polyethylene (PE) [14] and nanoplastics (NPs) [15]. Pharmaceuticals include ribavirin, azithromycin [13,16] and metformin [17].

**Figure 1:**
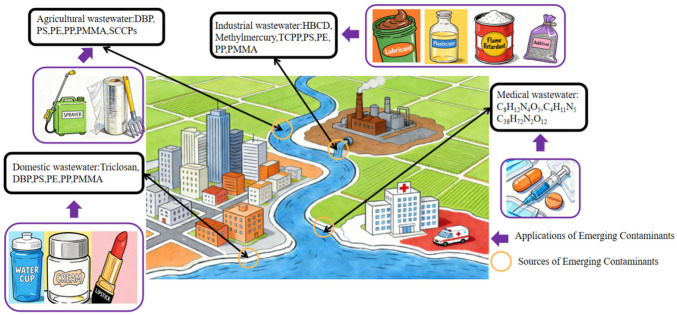
Primary Sources and Applications of Emerging Contaminants. Note: DBP = Dibutyl Phthalate; PE = Polyethylene; PP = Polypropylene; PS = Polystyrene; PMMA = Polymethyl Methacrylate; SCCPs = Short-Chain Chlorinated Paraffins; HBCD = Hexabromocyclododecane; TCPP = Tris (2-chloropropyl) Phosphate; C_8_H_12_N_4_O_5_ = Cytosine Arabinoside; C_4_H_11_N_5_ = 2,4,6-Triamino-1,3,5-Triazine; C_38_H_72_N_2_O_12_ = Microcystin-LR.

These emerging contaminants originate from diverse sources across industrial, domestic, medical, and agricultural sectors (Figure 1). For example, SCCPs are widely used as plasticizers, flame retardants, and lubricants in various industrial applications, and their widespread use leads to continuous release into the environment via industrial effluents, improper disposal, and atmospheric deposition [18]. In addition, triclosan is commonly found in personal care products such as cosmetics, deodorants, mouthwashes, and soaps, and it enters aquatic ecosystems primarily through domestic wastewater discharge [19]. Furthermore, chlorinated organophosphate esters and HBCDs, which act as flame retardants in plastics, textiles, and electronic devices, are released into the environment during manufacturing, product use, and waste recycling processes [20,21]. Likewise, microplastics including PMMA, PS, and PP mainly derive from the degradation and improper disposal of plastic materials [10,14,22]. Nanoplastics are subsequently formed via the further degradation of MPs [23], but they can also be produced directly [24]. Pharmaceutical contaminants such as azithromycin and metformin enter aquatic ecosystems through untreated or inadequately treated medical and domestic wastewater [25,26].

A growing body of evidence indicates that pharmaceuticals, EDCs, MPs, and other contaminants have been widely detected in marine waters, surface freshwater systems, and sediments worldwide, reflecting their high environmental persistence, extensive mobility, and significant ecological risks [27,28].

**Figure 2:**
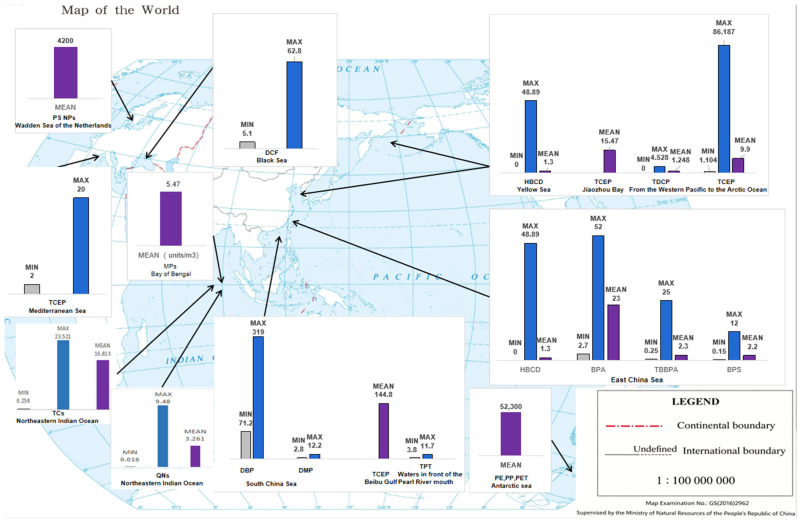
Global natural concentrations of emerging contaminants over the past five years. Note: This figure presents the concentrations (ng/L) of selected emerging contaminants in specific natural marine environments. The abbreviations are defined as follows: MPs = Microplastics; HBCD = Hexabromocyclododecane; TCEP = Tris (2-chloroethyl) Phosphate; TDCP = Tris (1,3-dichloro-2-propyl) Phosphate; BPA = Bisphenol A; BPS = Bisphenol S; TPT = Triphenyltin; TBBPA = Tetrabromobisphenol A; DBP = Dibutyl Phthalate; DMP = Dimethyl Phthalate; DCF = Diclofenac; PE = Polyethylene; PP = Polypropylene; PET = Polyethylene Terephthalate, TCs = Tetracycline Antibiotics; QNs = Quinolone Antibiotics.

A wide range of emerging contaminants have been detected in marine regions worldwide (Figure 2). The average concentration of HBCD was reported to be 1.30 ng/L in the Yellow Sea and East China Sea, while DBP concentrations ranged from 71.2 to 319 ng/L in the South China Sea. For TCEP, mean concentrations reached 9.9 ng/L in the Northwest Pacific and Arctic Ocean, varied between 2 and 20 ng/L in the Mediterranean Sea, peaked at 144.8 ng/L in the Beibu Gulf, and were 15.47 ng/L in Jiapzhou Bay in the East China Sea; average BPA concentrations ranged from 18 ± 9.7 ng/L to 23 ng/L. Furthermore, plastic-associated contaminants such as PE, PP, and PET were found at an average concentration of 52,300 ng/L in Antarctic sea ice. PS NPs were detected at 4200 ng/L in the Dutch Wadden Sea, while MPs were quantified at 5.47 units/m^3^ in the Bay of Bengal [20,26,29,30,31,32,33,34,35,36,37,38,39,40,41]. In the surface seawater of the Northeastern Indian Ocean, the concentration range of tetracycline antibiotics was 0.258–23.521 ng/L (mean: 16.813 ng/L), and that of quinolone antibiotics was 0.016–9.480 ng/L (mean: 3.261 ng/L) [42].

Marine invertebrates represent a core component of marine biodiversity and form the foundation of marine ecosystems. With tens of thousands of described species, they play pivotal roles in nutrient cycling, benthic structure formation, and food web connectivity, acting both as consumers of primary producers and as a critical food source for higher trophic-level predators [43,44]. Marine invertebrates are ideal model organisms for toxicological research due to their high species diversity, broad geographic distribution [45], ease of collection and culture, and low experimental costs. Most species exhibit short life cycles, allowing rapid assessment of both short- and long-term effects of toxicants [46]. Moreover, their high sensitivity to environmental pollutants enables precise elucidation of toxic mechanisms [1]. For taxa considered ecosystem keystone species, their toxicological responses can directly reflect ecological risks, granting them substantial scientific and ecological value [47].

The research objects selected in this review all belong to Arthropoda, Echinodermata and Mollusca. Species of Arthropoda have a short life cycle of approximately 3–4 months, with the advantages of fast reproduction, easy laboratory cultivation and simple experimental operation [43,44,46]. Sea urchins from Echinodermata serve as classic model organisms. Their embryonic development is easy to observe, and they possess a complete genome containing about 23,300 genes, covering almost all vertebrate gene families with low genetic redundancy. Moreover, they are highly sensitive to environmental pollutants, especially during early developmental stages [48]. Bivalves of Mollusca (such as mussels and oysters) are filter-feeding organisms that can actively accumulate emerging pollutants including heavy metals, pesticides and pharmaceutical residues from aquatic environments. The pollutant concentrations in their tissues can accurately reflect the actual pollution status of water bodies. In addition, their sessile lifestyle facilitates long-term monitoring and comprehensive environmental assessment. As typical pollution indicator organisms, they can effectively evaluate the overall pollution level of aquatic ecosystems. Meanwhile, bivalves occupy a crucial ecological niche in marine ecosystems and exert vital influences on ecosystem functions [49]. Therefore, organisms from these three phyla possess comprehensive superiorities and are regarded as the preferred experimental subjects for relevant environmental research.

The toxic effects of emerging contaminants reviewed in this paper on marine invertebrates are mainly reflected in two aspects: neurotoxicity and reproductive toxicity. Emerging particulate pollutants such as MPs can accumulate in the bodies of benthic or pelagic invertebrates, leading to a significant decrease in acetylcholinesterase (AChE) activity, neurotransmitter imbalance, and morphological damage to neural tissues [50,51,52,53]. In contrast, other emerging contaminants, including pharmaceutical residues and heavy metal complexes, can also induce neurobehavioral abnormalities (e.g., sluggish movement and weakened escape responses [54]) by interfering with neural signal transduction and triggering cell apoptosis [55]. These neurotoxic effects not only impair the survival and reproductive capacity of invertebrates but also transfer along the food chain, posing potential risks to higher trophic levels and even human health. The reproductive toxicity of emerging contaminants on marine invertebrates refers to the impairment of gametogenesis, fertilization, embryonic development, and offspring reproductive success after exposure, with adverse effects that may persist in subsequent generations or even across multiple generations [56,57,58]. Studies have shown that such contaminants can weaken the reproductive capacity of marine invertebrates through mechanisms including interfering with endocrine signaling, inducing oxidative stress, and altering gene expression, thereby threatening population maintenance and ecological functions [59]. For instance, in sea cucumbers, *Tigriopus japonicus*, and marine mussels, *Mytilus edulis*, BPA, MPs and various antibiotics have been shown to significantly reduce the production of male and female gametes, delay oocyte maturation, and cause reproductive organ malformations; some of these effects remain detectable in multi-generational experiments [60,61,62,63]. In *Mytilus galloprovincialis*, individual or combined exposure to silver nanoparticles, polystyrene nanoparticles, and pharmaceuticals such as 5-fluorouracil can disrupt gonadal enzyme activity, exacerbate oxidative damage, and consequently reduce fertilization rates and larval survival rates [64]. These findings indicate that such contaminants exert direct and persistent inhibitory effects on the reproductive functions of bivalves. Numerous studies have demonstrated that research efforts have mostly focused on single-contaminant exposure and single-generational effects, whereas the ecological impacts of combined exposure remain insufficiently explored. At present, comprehensive studies integrating the toxic effects of emerging contaminants on marine invertebrates—especially those involving combined exposure—are relatively scarce. Therefore, while elaborating on the toxic effects of individual emerging contaminants on marine invertebrates, this paper also summarizes the toxic impacts of combined exposure reported in the past five years, thereby providing a crucial reference for future research and exploration in marine ecotoxicology.

## 2. Methodology

This systematic review was conducted in strict adherence to the PRISMA guidelines to ensure rigor, transparency, and critical evaluation throughout the assessment process. Using a structured literature screening workflow, a multi-dimensional analytical framework, and transparent methodologies, the study not only precisely identified current research gaps in the field but also enhanced the credibility of conclusions and the academic value of the findings [65]. It systematically synthesized existing research on the reproductive and neurotoxic effects of emerging contaminants in marine invertebrates. During the study, relevant toxic effects were clearly elucidated, with a focus on analyzing interspecies sensitivity differences and exploring underlying toxic mechanisms, while also explicitly identifying limitations in current research. In the literature search phase, the study clearly defined search databases (e.g., Web of Science https://www.webofscience.com/wos/, accessed on 16 May 2026, CNKI https://www.cnki.net/, accessed on 16 May 2026, PubMed https://pubmed.ncbi.nlm.nih.gov/, accessed on 16 May 2026), a time frame (2020–2025), and bilingual search term combinations, with the search focus directed toward the neurotoxic and reproductive effects of emerging contaminants in marine invertebrates. A comprehensive four-step process was employed for literature screening: duplicate removal, automated initial screening, manual initial screening, and full-text evaluation. This meticulous and rigorous screening approach ensured the relevance and scientific rigor of included studies and fully complied with the transparency standards of the PRISMA guidelines (see Figure 2). Additionally, the study conducted literature searches in both English and Chinese databases using core keywords: emerging contaminants, marine invertebrates, neurotoxicity, reproductive toxicity, and environmental concentrations. A comprehensive evaluation framework was developed by integrating the CRED guidelines and EPA toxicity assessment criteria [66,67], classifying the quality of included studies into three levels: high, moderate, and low.

## 3. Toxicological Impacts of Single Emerging Contaminant Exposure on Marine Invertebrates

### 3.1. Impacts of Reproductive Toxicity

Emerging contaminants exert significant reproductive toxicity on a variety of invertebrates, which is primarily manifested by disrupting key physiological processes such as gonadal development, oocyte maturation and embryogenesis (Table 1). Among these contaminants, HBCD, BBP and TBDE all exhibit distinct reproductive inhibitory effects on *Brachionus plicatilis*, EC_50_ values typically ranging from 0.5 to 0.8 mg/L. The underlying toxic mechanisms mainly involve oxidative stress, apoptosis, and aberrant gene expression [58,59,60,61,62,63,64,65,66,67,68,69,70]. Notably, *B. plicatilis*, an arthropod species, is particularly sensitive to contaminants including HBCD and BBP. For some of these pollutants, the EC_50_ values are below 0.5 mg/L, and the NOEC can be as low as 0.1 mg/L. For TPT, the lowest reported NOEC for reproductive toxicity is 10 ng/L, a concentration that is well within the range of TPT levels measured in surface waters. This means that environmentally relevant TPT exposures, even at concentrations currently present in the field, have the potential to induce reproductive inhibition and ovarian damage in this species. Such findings underscore the hazard of TPT as an endocrine-disrupting chemical, even at the low levels encountered in the environment [68,69,70,71]. In mollusks, contaminants such as BPA and PFOA can impair the reproductive functions of mussels by interfering with estrogen receptors and downregulating genes related to steroid metabolism, with the LOEC as low as 693 μg/L [72,73]. Specifically, even at a concentration as low as 2 μg/L, PFOA exerts measurable toxic effects on *Mytilus coruscus*: downregulating the expression of steroid metabolism genes, disrupting gonadal structure, reducing sperm motility, and causing abnormal oocyte development. Thus underscoring the high hazard potential of such contaminants [73]. Echinoderms are not exempt from the harmful effects of emerging contaminants. Exposure of *Strongylocentrotus purpuratus* to CuO NPs and MPs disrupts embryonic development at multiple levels: it inhibits secondary mesenchyme cell migration, interferes with nerve conduction and skeletogenesis, and induces exudate-related skeletal malformation and nerve cell damage, collectively resulting in skeletal deformities and neurodevelopmental defects [54,74]. Overall, relatively low concentrations of emerging contaminants can cause measurable damage to the reproductive systems of invertebrates, suggesting that these pollutants pose considerable potential ecological risks in the environment. Their long-term cumulative effects and multi-generational impacts urgently require further in-depth investigation.

### 3.2. Impacts of Neurotoxicity

A variety of emerging contaminants exhibit significant neurotoxic effects on invertebrates, primarily acting through mechanisms such as disrupting neurotransmitter systems, inhibiting the activity of key enzymes, and inducing oxidative stress (Table 1). For instance, antibiotics detected in *Mytilus galloprovincialis*, including Ciprofloxacin (CIP), Enrofloxacin (ENR) and Danofloxacin (DAN), can inhibit acetylcholinesterase (AChE) activity and interfere with neural signal transduction, with the LOEC as low as 5–6 ng/L, indicating extremely high sensitivity of this species. These antibiotics exert their neurotoxicity mainly by suppressing acetylcholinesterase activity [53]. PFECA and PFOA disrupt embryonic neural development and skeletal formation in *Hemicentrotus pulcherrimus*, with EC_50_ values of 0.05 mg/L and 0.20 mg/L, respectively, at the early developmental stage [75]. In addition, BPA induces behavioral inhibition, neuronal apoptosis, and changes in membrane fluidity in rotifers, with an LOEC of 0.5 mg/L [76]. These findings suggest that even at relatively low environmental concentrations, such contaminants may pose potential hazards to the nervous systems of aquatic invertebrates.

**Table 1:** Toxicological responses of marine invertebrates to single emerging contaminant exposure.

Emerging Contaminants	Phyla	Experimental Subjects	Toxicity	Toxicological Effects	Effect Concentration	References
**CIP**	Mollusca	*M. galloprovincialis*	Neurotoxicity	Inhibit AChE.	LOEC: 5 ng/L	[53]
**ENR**	Mollusca	*M. galloprovincialis*	Neurotoxicity		LOEC: 6 ng/L	
**DAN**	Mollusca	*M. galloprovincialis*	Neurotoxicity		NOEC: 5 ng/L LOEC: 50 ng/L	
**CuO NPs**	Echinodermata	*S. purpuratus*	Reproductive toxicity	Inhibit the migration of secondary mesenchyme cells and interfere with nerve conduction and skeletogenesis.	EC_50_ (sperm toxicity): 0.06 mg/L EC_50_ (embryonic development): 5395 ppb	[54]
**HBCD**	Arthropoda	*B. plicatilis*	Reproductive toxicity	Induce oxidative stress and apoptosis, and impair ovarian development.	EC_50_ (chronic exposure): 0.8 mg/L NOEC: 0.2 mg/L	[68]
**BBP**	Arthropoda	*B. plicatilis*	Reproductive toxicity	Induce the production of reactive oxygen species (ROS), activate the endoplasmic reticulum stress pathway, inhibit the activity of antioxidant enzymes and ultimately lead to ovarian cell apoptosis.	Dose-dependent reproductive inhibition: 0.1–10 mg/L Significant reproductive inhibition: 0.001–1 mg/L	[69]
**BDE-47**	Arthropoda	*B. plicatilis*	Reproductive toxicity	Induce oxidative stress, inhibit the expression of *Vasa* and *Nanos* genes and cause ovarian damage.	EC_50_ (acute exposure): 0.5 mg/L NOEC (chronic exposure): 0.1 mg/L	[70]
**TPT**	Arthropoda	*B. plicatilis*	Reproductive toxicity	Induce oxidative damage and dysregulation of the *Vasa* gene, thereby delaying the reproductive peak.	NOEC: 10 ng/L LOEC (spawning inhibition): 100 ng/L	[71]
**PFOA**	Mollusca	*M. coruscus*	Reproductive toxicity	Downregulate the expression of steroid metabolism genes, disrupt gonadal structure, reduce sperm motility, and cause abnormal oocyte development.	Experimental concentrations: 2–200 μg/L	[73]
**MPs**	Echinodermata	*S. purpuratus*	Reproductive toxicity	The exudate induces embryonic skeletal malformation and impairs the development of nerve cells.	LC_50_ (embryonic mortality): >10 mg/L	[74]
**BPA**	Arthropoda	*B. plicatilis*	Neurotoxicity	Inhibition of swimming behavior and disturbance of neurotransmitter homeostasis.	LOEC: 0.5 mg/L	[75]
**PFECA**	Echinodermata	*H. pulcherrimus*	Neurotoxicity	Disrupt dorsoventral (DV) axis specification, reduce the differentiation of endoderm and secondary mesenchyme cells, and impair neural development.	EC_50_ (Early-stage): 0.05 mg/L EC_50_ (Late-stage): 0.15 mg/L	[76]
**PFOA**	Echinodermata	*H. pulcherrimus*	Neurotoxicity	Disrupt the expression of skeletal patterning genes, leading to skeletal malformation; exert effects later than PFECA.	EC_50_ (Early-stage): 0.20 mg/L EC_50_ (Late-stage): 0.80 mg/L	
**TCEP**	Arthropoda	*T. japonicus*	Reproductive toxicity	Oxidative stress, mitochondrial dysfunction, and altered gene expression.	NOEC (chronic exposure): 0.892 mg/L	[77]
**TPT**	Mollusca	*Perna viridis*	Reproductive toxicity	Target the RXR nuclear receptor and inhibit the steroid biosynthesis gene (*hsd17b*).	LC_50_ (96 h): 18.7 μg/L EC_50_ (96 h): 2.7 μg/L	[78]
**THI**	Arthropoda	*Litopenaeus vannamei*	Reproductive toxicity	Developmental toxicity, affecting larval survival rate and neural development.	LC_50_ (48 h): 175 μg/L	[79]
**TDCPP**	Mollusca	*M. galloprovincialis*	Neurotoxicity	AChE inhibition leads to blocked synaptic signal transmission and downregulation of *PTPRN2*.	NOEC: 10 μg/L LOEC (significant decrease in AChE activity): 10 μg/L	[80]

Note: HBCD = Hexabromocyclododecane; BBP = Butyl Benzyl Phthalate; BDE-47 = Tetrabromodiphenyl Ether 47; TPT = Triphenyltin; CuO NPs = Copper Oxide Nanoparticles; TCEP = Tris(2-chloroethyl) Phosphate; PFOA = Perfluorooctanoic Acid; MPs = Microplastics; THI = Thiamethoxam; CIP = Ciprofloxacin; ENR = Enrofloxacin; DAN = Danofloxacin; PFECA = Perfluoroalkyl Ether Carboxylic Acids; TDCPP = Tris (1,3-dichloro-2-propyl) Phosphate; BPA = Bisphenol A. LC_50_ = median lethal concentration; EC_50_ = median effective concentration; NOEC = no-observed-effect concentration; LOEC = lowest observed effect concentration.

### 3.3. Summary of Toxicity Comparisons

The concentration range of toxic impacts of emerging contaminants on invertebrates spans from mg/L to ng/L (Table 1), thus exhibiting the characteristics of low-dose exposure and high biological sensitivity. Among these organisms, Arthropoda (e.g., *B. plicatilis*, *Tigriopus japonicus*) demonstrates high vulnerability in both reproductive and neurotoxicological responses. This highlights the need to prioritize this taxonomic group and its corresponding contaminant exposure thresholds in ecological risk assessments. Notably, significant differences exist in the toxic effects of a single contaminant across different species. For example, PFOA induces reproductive damage in *M. coruscus* [73], while it primarily disrupts neural development and skeletal formation in *H. pulcherrimus* [75]. These findings indicate that the ecological toxicity of emerging contaminants is a species-specific and complex outcome. Their ultimate impacts depend not only on the chemical properties of the contaminants themselves but also on the combined modulation of multiple factors, including the test organism’s species, physiological state, life history stage, and environmental media (e.g., salinity, temperature, coexisting contaminants) [81,82]. Current research primarily focuses on model organisms or specific taxonomic groups, but the sample coverage and environmental realism remain insufficient. Therefore, to develop exposure-effect models capable of accurately predicting the toxic intensity of contaminants in complex real-world environments, future studies must adopt more systematic, multi-species and multi-scenario integrated research approaches. This will help supplement the data foundation and validate the generality and reliability of such models.

## 4. Toxicological Impacts of Combined Emerging Contaminant Exposure on Marine Invertebrates

### 4.1. Impacts of Reproductive Toxicity

In terms of reproductive toxicity, significant synergistic effects often occur when contaminants act on the reproductive system through similar or complementary mechanisms, thereby exacerbating reproductive damage. For example, when *Arbacia lixula* is co-exposed to BPA and BPS, the combined LC_50_ drops to <10 mg/L, which is much lower than the >100 mg/L observed in single-contaminant exposure (Table 2). Both contaminants significantly amplify reproductive toxicity by jointly interfering with steroid hormone receptors, disrupting embryonic cell division, and increasing chromosomal abnormalities [83]. Similarly, co-exposure of *Artemia salina* to TBBPA and Bz also exhibits synergistic effects, which damage the mitochondrial respiratory chain and inhibit ATPase activity, leading to energy metabolism collapse [84]. However, the involvement of MPs may reduce the bioavailability of contaminants by means of physical adsorption or activation of biological detoxification systems such as the MXR pathway, thus exerting antagonistic effects and alleviating reproductive toxicity. For instance, when rotifers are co-exposed to PS MPs with TCEP or TBT, MPs accumulate in the digestive tract and upregulate the expression of ABC transporters and heat shock proteins, enhancing contaminant efflux and mitigating the population growth inhibition and oxidative stress induced by TCEP or TBT [85,86]. In addition, combined reproductive toxicity is also concentration-dependent. For example, the combined exposure to BDE-47 and DBDPE shows an antagonistic effect at low concentrations, but shifts to a synergistic effect at moderate to high concentrations [87]. These findings indicate that in the real marine environment, the ultimate impact of co-existing contaminants on the reproductive function of invertebrates is a dynamically modulated outcome governed by multiple factors, and cannot be simply inferred based on single-contaminant toxicity data.

### 4.2. Impacts of Neurotoxicity

The neurotoxicity of combined contaminant exposure to marine invertebrates usually exhibits a significant synergistic enhancement effect, with the toxic intensity far exceeding the simple superposition of the effects of individual contaminants. When contaminants with distinct mechanisms of action act jointly, they can cause multiple damages to the nervous systems of marine invertebrates, thereby producing a superadditive potentiation. Such synergistic effects have been fully verified in relevant exposure experiments: when PS-NPs and CBZ act in combination on *M. edulis*, PS-NPs not only inhibit the activity of AChE by themselves, but also adsorb CBZ through the “Trojan horse” effect, significantly increasing its bioavailability in organisms and thus synergistically exacerbating neurotoxicity [88,89]. Co-exposure of NEOs and MPs to *M. edulis* prolongs the retention time of contaminants in biological tissues, interferes with the expression level of GABAergic receptors, and reduces neuronal excitability. The EC_50_ of their combined action is as low as 0.09 μg/L, showing strong synergistic toxicity [90]. Co-exposure of permethrin and PS-NPs to *A. salina* leads to a sharp increase in intracellular ROS levels by up to 87.94%, triggering severe oxidative damage, accompanied by a significant decrease in swimming ability and movement speed. Moreover, the mixed exposure of CQ and Cu to *Proales similis* induces cross-toxicity by interfering with mitochondrial respiratory chain function and inducing oxidative stress. All concentration combinations exhibit synergistic or even extremely strong synergistic effects, which are specifically reflected in the significant decrease in population growth rate (r) with the increase in exposure concentration. In the high-concentration exposure group (M4), the growth rate even drops to a negative value [91,92].

**Table 2:** Toxicological responses of marine invertebrates to combined emerging contaminant exposure.

Emerging Contaminants	Phyla	Experimental Subjects	Toxicity	Single Exposure Concentration	Combined Exposure Concentration	Toxicological Effects	Joint Action	References
**CBZ + PS**	Mollusca	*M. galloprovincialis*	Neurotoxicity	EC_50_ (CBZ): 45 µg/L LC_50_ (PS-NPs): 3.2 mg/L	0.28 mg/L (PS) + 6 µg/L (CBZ)	Nanoplastics act as carriers to enhance the bioavailability of CBZ; exert dual inhibitory effects on AChE and glutamic acid decarboxylase (GAD); and significantly exacerbate oxidative damage as reflected by elevated malondialdehyde (MDA) levels.	Synergism	[1]
**BPA + BPS**	Echinodermata	*A. lixula*	Reproductive toxicity	LC_50_ (BPA): >100 mg/L LC_50_ (BPS): >100 mg/L	LC_50_ (Combined): <10 mg/L	Interfere with steroid hormone receptors; disrupt early embryonic cell division; and jointly increase the rate of chromosomal abnormalities.	Synergism	[83]
**TBBPA + Bz**	Arthropoda	*A. salina*	Reproductive toxicity	LC_50_ (TBBPA): 17.05 μg/L LC_50_ (BZ): 14.86 mg/L	LC_50_ (Combined): <10 μg/L	Disrupt the mitochondrial respiratory chain; inhibit ATPase activity; co-exposure leads to energy metabolism collapse.	Synergism	[84]
**PS-MPs + TCEP**	Arthropoda	*B. plicatilis*	Reproductive toxicity	LC_50_ (TCEP): 672 mg/L	Exposure Concentration: 1/10 LC_50_: 6.5 mg/L	Microplastics accumulate in the digestive tract, activate the multixenobiotic resistance (MXR) system, upregulate ABC transporters (P-gp) and heat shock proteins, thereby promoting xenobiotic efflux, reducing intracellular TCEP levels, and alleviating TCEP-induced growth inhibition and oxidative stress. Notably, 1 μm microplastics significantly reversed these toxic effects.	Antagonism	[85]
**TBT + PS-MPs**	Arthropoda	*Brachionus koreanus*	Reproductive toxicity	PS-MPs: 10 µg/L	—	Microplastics or high-nutrient feed mitigate TBT toxicity by reducing its uptake or enhancing metabolic detoxification (e.g., upregulating GST and heat shock proteins). At 10 µg/L (PS MPs), they significantly alleviated TBT-induced reproductive inhibition.	Antagonism	[86]
**BED-47** **+ DBDPE**	Arthropoda	*B. plicatilis*	Reproductive toxicity	—	NOEC = 5 µg/L (no significant inhibition was observed under either single or combined exposure), LOEC: 50 µg/L (reproductive inhibition occurred). Antagonism was exhibited at low concentrations (5 µg/L); synergism was exhibited at medium to high concentrations (50–500 µg/L), with the inhibition rate increasing significantly.	Co-exposure to two PBDE congeners results in decreased activities of energy metabolism enzymes (α-amylase, lipase, trypsin), reduced filtration/feeding rates, total ATP depletion, lowered fertilization rates, decreased total larval counts, and shortened reproductive periods.	Antagonism/Synergism	[87]
**CBZ + PS-NPs**	Mollusca	*H. pulcherrimus*	Neurotoxicity	PS-NPs: 0.05 mg/L CBZ: 6.3 µg/L	0.05 mg/L (PS-NPs) + 6.3 µg/L (CBZ)	PS-NPs significantly inhibit AChE activity; moreover, PS-NPs adsorb CBZ via the “Trojan horse” effect, alter its bioavailability, and enhance neurotoxicity.	Synergism	[88]
**NEOs + MPs**	Mollusca	*H. pulcherrimus*	Neurotoxicity	NEOs: 0.1 µg/L; MPs: 1 mg/L	NEOs: 0.1 µg/L + MPs: 1 mg/L EC_50_ (Combined): 0.09 µg/L	Microplastics adsorb neonicotinoids, prolong their retention time in tissues, induce upregulation of GABAergic receptor expression, and reduce neuronal excitability.	Synergism	[90]
**CQ+ Cu**	Arthropoda	*P. similis*	Neurotoxicity	24 h LC_50_(CQ): 4.25 mg/L 24 h LC_50_ (Cu): 68 μg/L	**CQ concentrations**: M1: 212.5 μg/L (AF = 0.05) M2: 425 μg/L (AF = 0.1) M3: 1275 μg/L (AF = 0.3) M4: 2125 μg/L (AF = 0.5) **Cu concentrations**: M1: 3.5 μg/L (AF = 0.05) M2: 7 μg/L (AF = 0.1) M3: 20 μg/L (AF = 0.3) M4: 34 μg/L (AF = 0.5)	Cu and CQ may interfere with the mitochondrial respiratory chain (e.g., blocking electron transfer), leading to impaired energy production. Cu-induced oxidative stress (ROS generation) may exert cross-toxicity with the biotransformation process of CQ, amplifying the damage to organisms. M1: CQ: r = 0.39 ± 0.01 d^−1^, Cu: r = 0.42 ± 0.01 d^−1^ M2: CQ: r ≈ 0.34 d^−1^, Cu: r ≈ 0.34 d^−1^ M3: CQ: r ≈ 0.28 d^−1^, Cu: r = 0.21 ± 0.01 d^−1^ M4: CQ: r ≈ 0.19 d^−1^, Cu: r = 0.09 ± 0.02 d^−1^ **Population growth rate (***r***)**: M1: *r* = 0.29 ± 0.01 d^−1^ (synergism) M2: *r* = 0.20 ± 0.01 d^−1^ (synergism) M3: *r* = 0.09 ± 0.02 d^−1^ (strong synergism) M4: *r* = −0.03 ± 0.01 d^−1^ (extremely strong synergism)	Synergism	[91,92]
**Permethrin + PS-NPs**	Arthropoda	*A. salina*	Neurotoxicity	LC_50_ (Permethrin): 4.536 mg/L	LC_50_ (Combined): 3.127 mg/L	The ROS level increased by 87.94%, causing cellular oxidative damage, significantly reducing swimming ability and locomotor speed, impairing the body surface structure, and affecting normal physiological functions.	Synergism	[93]
**4-NPEs +PS-NPs**	Arthropoda	*A. salina*	Reproductive toxicity	LC_50_ (NPER): 4.5 mg/L LC_50_ (PS-NPs): >10 mg/L	LC_50_ (Combined): 3.1 mg/L	NPER induces ROS outburst; PS-NPs adsorb heavy metals; co-exposure leads to membrane lipid peroxidation and morphological aberration.	Synergism	[93]
**PE MPs + CET+ BCP**	Mollusca	*M. galloprovincialis*	Neurotoxicity	—	500 ng/L (CIT) + 500 ng/L (BEZ) + 1 mg/L (PE MPs)	Oxidative damage is enhanced; cholinergic neurotransmission is disrupted; metabolomic profiles are significantly altered. Studies have found that co-exposure results in higher bioaccumulation and toxicity than single exposure.	Synergism	[94,95]

Note: PS-NPs = Polystyrene Nanoplastics; BPA = Bisphenol A; BPS = Bisphenol S; 4-NPEs = 4-Nonylphenol Ethoxylates; TBBPA = Tetrabromobisphenol A; Bz = Benzene; PS-MPs = Polystyrene Microplastics; TBT = Tributyltin; DBDPE = Decabromodiphenyl Ethane; CBZ = Carbamazepine; MPs = Microplastics; NEOs = Neonicotinoid Insecticides; CQ = Chloroquine; Cu = Copper; PS = Polystyrene; PE MPs = Polyethylene Microplastics; CET = Cetirizine; BCP = Benzyl Chlorophenol. LC_50_ = median lethal concentration; EC_50_ = median effective concentration; NOEC = no-observed-effect concentration; LOEC = lowest observed effect concentration; M1–M4 = experimental groups; r = population growth rate; AF = concentration factor (the concentration gradient was determined via the LC_50_ multiplication method (AF method)).

## 5. Joint Toxicological Action of Combined Emerging Contaminant Exposure on Marine Invertebrates

Based on the combined toxicity data of emerging contaminants presented in Table 2, their joint action exhibits a complex pattern involving synergism, antagonism and concentration dependence, leading to ecological risks to invertebrates that far exceed the simple superposition of individual contaminants. Synergism represents the predominant manifestation and is widely observed in both reproductive and neurotoxicity endpoints. Three typical contaminant combinations consistently induce significant synergistic effects. The first type involves pollutants with distinct mechanisms of action, such as TBBPA mixed with Bz [84]. The second includes structurally analogous contaminants (e.g., BPA and BPS) [83]. The third refers to systems where microplastics act as carriers. They enhance the bioavailability of other pollutants, such as CBZ combined with PS [1]. In contrast, antagonistic effects are mostly associated with physical sequestration of pollutants or activation of biological detoxification mechanisms. A typical example is the co-exposure of PS-MPs with TCEP or TBT. PS-MPs can either reduce the aqueous bioavailable concentration of co-existing pollutants through adsorption or upregulate the MXR system to enhance contaminant efflux, thereby mitigating toxicological impacts. However, such antagonistic effects are highly uncertain and may potentially mask the long-term cumulative risks posed by these contaminants [85,86]. Combined toxicity also displays pronounced concentration dependence. For instance, the mixture of BDE-47 and DBDPE exerts an antagonistic effect at low concentrations (5 µg/L) but shifts to synergism at moderate to high concentrations (50–500 µg/L) [87]. Another illustrative case is the M4 treatment group of CQ plus Cu (CQ: 2125 μg/L; Cu: 34 μg/L), where the population growth rate reached its minimum and even turned negative, demonstrating extremely strong synergistic toxicity [91]. These findings highlight that environmentally realistic concentrations are critical yet underexplored for assessing the joint risks of emerging contaminants. As shown in Figure 1 and Table 2, most laboratory experiments use concentrations far exceeding those in natural marine environments. Mixture toxicity is highly concentration-dependent: combinations like BDE-47/DBDPE exhibit antagonism at low, environmentally relevant concentrations but shift to synergism at elevated levels, which limits extrapolation of lab-derived risk assessments to real ecosystems. Notably, a five-year literature review reveals a severe lack of studies on multi-contaminant (≥3) co-exposure at environmentally realistic concentrations. This gap is significant, as mixture toxicity to marine invertebrates involves dynamic synergistic/antagonistic interactions modulated by interactive biotic and abiotic factors, complicating risk extrapolation from simplified laboratory conditions.

A growing body of research has identified environmental context as one of the key determinants governing the nature of combined effects (i.e., antagonism or synergism), encompassing external physicochemical factors such as pollutant mixture concentration, ratio, temperature, and pH [96,97]. For instance, co-exposure to BDE-47 and dibromodiphenyl ether (di-BDE) has been shown to exhibit predominantly antagonistic effects at low concentrations, whereas the interaction shifts to significant synergism at medium to high concentrations, highlighting the core influence of concentration dependence [87]. Furthermore, the type and intensity of toxicity induced by combined exposure are highly combination-specific. When a single emerging pollutant (e.g., MPs) is co-administered with different contaminants such as TCEP [85], TBT [86], and CBZ [1], drastically divergent toxic outcomes—ranging from strong antagonism to marked synergism—may arise through entirely distinct mechanisms. These mechanisms include reduced bioavailability via physical adsorption, enhanced bioaccumulation through carrier effects, and activation of biological detoxification systems. As a case in point, PS-NPs exert an antagonistic effect with TCEP in rotifers [85], whereas they act synergistically with CBZ in mussels [1]. This discrepancy hinges on the role played by MPs in specific combinations, namely whether they function as a toxicity-mitigating carrier or a toxicity-amplifying carrier. Therefore, current toxicological datasets derived from single pollutants or simple binary mixtures are far from adequate to enable accurate prediction and assessment of the composite ecological risks posed by multiple pollutants in real marine environments. Moving forward, it is imperative to systematically design and conduct complex exposure experiments incorporating multiple pollutants, multi-concentration gradients, and diverse environmental factors. Such efforts will yield more comprehensive and environmentally realistic toxicological data, thereby laying a robust scientific foundation for the development of reliable composite pollution risk assessment models.

## 6. Toxic Mechanisms of Emerging Contaminants on Marine Invertebrates

### 6.1. Toxic Mechanisms of Individual Emerging Contaminants on Marine Invertebrates

Under single-exposure conditions, contaminants exert their toxic effects primarily through pathways including oxidative stress induction, gene expression disruption, key enzyme activity inhibition and endocrine signaling interference (Table 1). For example, HBCD induces excessive production of ROS, activates calcium signaling to amplify toxic effects and simultaneously causes a significant increase in DNA fragmentation and apoptosis, thereby exacerbating reproductive inhibition. BBP rapidly triggers oxidative stress at different concentrations, characterized by a marked elevation in superoxide dismutase (SOD) activity, and disrupts lipid metabolism, leading to a reduction in malondialdehyde (MDA) content [68]. Furthermore, BBP severely damages the endoplasmic reticulum (ER) structure of female reproductive organs. Transcriptome analysis reveals significant alterations in the expression of genes associated with redox regulation, biosynthetic processes, and cellular damage, which further exacerbates ER stress and impairs cellular functions, ultimately resulting in reproductive toxicity [69]. TBDE disrupts purine and pyrimidine metabolism in *B. plicatilis*, leading to an imbalance between nucleotide synthesis and degradation. The downregulated expression of glutamine synthetase (GS) and increased activity of xanthine oxidase (XOD) promote excessive ROS generation, which in turn induces DNA oxidative damage (evidenced by elevated 8-hydroxy-2′-deoxyguanosine (8-OHdG) levels). This activates the p53 signaling pathway, upregulates the expression of p53 and Bax, downregulates Bcl-2, increases the Bax/Bcl-2 ratio, and ultimately triggers cellular apoptosis [70]. Quinolone antibiotics, such as CIP, significantly inhibit the activities of glutathione S-transferase (GST) and AChE in the digestive glands of mussels at high concentrations, while also causing DNA single-strand breaks. ENR reduces protein carbonyl content in the gill tissues of mussels, and simultaneously induces increased glutathione peroxidase activity and AChE inhibition in the digestive glands. DAN impairs antioxidant enzyme activity and induces DNA damage at high concentrations [53].

### 6.2. Toxic Mechanisms of Combined Emerging Contaminants on Marine Invertebrates

In combined exposure scenarios, interactions among contaminants further amplify toxic effects, with synergism predominating (Table 2). Taken together, co-occurring contaminants frequently exert more severe toxicity than single substances, driven by diverse molecular interactions. For instance, BPA significantly induces embryo malformation in sea urchins at concentrations ranging from 0.25 to 100 µM, and triggers cytogenetic toxicity at 25 µM and 100 µM, characterized by decreased mitotic activity, chromosomal aberrations, and DNA damage. The underlying mechanisms may involve estrogen receptor activation, oxidative stress induction and epigenetic modifications. In contrast, BPS causes embryo mortality and morphological abnormalities within the same concentration range, yet no chromosomal abnormalities or mitotic inhibition were observed [83]. These findings indicate that BPS exhibits weak cytogenetic toxicity, with its primary toxic endpoint being the disruption of embryonic development. Nevertheless, the co-exposure of BPA and BPS significantly enhances reproductive toxicity in *A. lixula* through the combined disruption of steroid hormone receptors and impairment of cell division [83]. MPs play a dual role in combined toxicity. On one hand, they can act as carriers to increase the bioavailability of other contaminants (e.g., CBZ), and induce neurofunctional impairment by triggering oxidative stress, disrupting neurotransmitter homeostasis, activating the p38 and MAPK signaling pathways, and upregulating stress-related genes [1]. Microplastics can also adsorb neonicotinoids, prolonging their tissue retention time, which leads to the upregulation of GABAergic receptor expression, reduced neuronal excitability, and exacerbated neurotoxicity [90]. On the other hand, microplastics can alleviate toxic manifestations by adsorbing contaminants or activating the MXR system. For example, co-exposure to microplastics and TCEP causes population growth inhibition and elevated oxidative stress in *B. koreanus*. However, after the enrichment of 1 µm PS MPs in the digestive tract, the expression of ABC transporters and heat shock proteins is upregulated, enhancing P-glycoprotein efflux activity. This restores oxidative stress levels and growth inhibition to normal ranges, thereby mitigating toxic effects [85]. Additionally, exposure to TBT significantly increases ROS levels in *B. koreanus*, disrupts the antioxidant system, inhibits reproduction, and shortens lifespan. However, under conditions of sufficient food supply (high-dose diet) or co-exposure with microplastics, TBT-induced ROS accumulation can be partially reduced and antioxidant capacity restored, thereby alleviating its reproductive toxicity [86]. Cu and CQ may interfere with the mitochondrial respiratory chain (e.g., by blocking electron transport), leading to impaired energy production. Oxidative stress induced by Cu (via ROS generation) may exert cross-toxicity with the biotransformation process of CQ, amplifying damage to organisms [91,92]. Overall, these examples demonstrate that mixture toxicity is highly complex, shaped by contaminant type, exposure route, and species-specific responses.

## 7. Conclusions and Outlook

Widely detected in global aquatic environments, emerging contaminants primarily consist of POPs, EDCs, pharmaceuticals, and microplastics. Originating from industrial, agricultural, medical, and daily human activities, these pollutants feature persistence, bioaccumulation, and long-distance migration, thereby posing potential threats to marine ecosystems. Marine invertebrates, as key foundational organisms in marine systems, are highly sensitive to low-dose contaminant exposure, with predominant toxic responses including reproductive and neurotoxic damage. Considering the complex co-occurrence of various pollutants in actual aquatic environments, this study proposes optimized experimental protocols for field sample toxicity evaluation using marine invertebrate bioassays: environmentally realistic concentrations are applied to simulate natural low-exposure scenarios; multi-trophic invertebrate species are selected to reflect biological sensitivity toward mixed pollutants; combined reproductive and neurotoxic biomarkers are monitored to identify sublethal adverse effects; and rigorous sample pretreatment, blank controls, and biological replicates are utilized to reduce environmental interference. Such optimized strategies effectively improve the accuracy and ecological representativeness of bioassays, enabling reliable risk assessment of complex emerging contaminant mixtures in practical marine environments.

Emerging contaminants (including POPs, EDCs, pharmaceuticals and MPs) primarily induce reproductive and neurotoxicity in marine invertebrates, featuring the characteristics of low-dose effectiveness and high biological sensitivity. Under single-exposure conditions, arthropods (e.g., rotifers and cladocerans) exhibit extremely high vulnerability to various contaminants, with their EC_50_ and LOEC values often as low as the µg/L or even ng/L level. Significant differences exist in the toxic effects and underlying mechanisms of the same contaminant (e.g., BPA and PFOA) across different species, reflecting distinct species specificity. In-depth elucidation of the action mechanisms of individual contaminants not only facilitates the identification of contaminant categories with the most prominent impacts on marine ecosystems, thereby guiding source reduction, but also provides novel insights for fields such as medicine and materials science (e.g., the interference mechanisms of certain contaminants on specific biological pathways may be translated into valuable bioregulatory tools). More notably, multiple contaminants coexisting in the environment can exert complex combined toxic effects, which are mainly manifested in two modes: synergism and antagonism. When contaminants act jointly through different mechanisms (e.g., endocrine disruption, oxidative stress induction and key enzyme activity inhibition), strong synergistic effects tend to occur, implying hazards far exceeding those of individual contaminant toxicity. In particular, particulate matter such as MPs plays a dual role in combined exposure scenarios, it can act as a carrier that exacerbates the bioaccumulation and toxicity of other pollutants (synergism), or mitigates toxicity via adsorption of contaminants or activation of biological detoxification systems (antagonism). Additionally, combined toxicity exhibits distinct concentration dependence, where the same mixture may exert opposite effects at different concentration levels.

Current research still presents obvious limitations: most studies focus on single or binary contaminant exposure experiments, while research on ternary and higher-order complex pollution, as well as the interactive effects of multiple environmental factors (e.g., temperature and salinity) in real-world settings, remains severely insufficient, leading to substantial data gaps. Therefore, future ecotoxicological research urgently needs to expand toward more systematic composite exposure experiments incorporating multiple contaminants, multi-concentration gradients, and diverse environmental scenarios. Concurrently, it is essential to strengthen the observation of long-term and multi-generational toxic effects. These efforts will facilitate the construction of reliable assessment models that can accurately predict the combined ecological risks of emerging contaminants in real marine environments, thereby providing a solid scientific basis for marine environmental protection and ecological safety management.

## Data Availability

No new data were created or analyzed in this study. Data sharing is not applicable to this article.
